# Supplementary material for: Conventional and antibody-enhanced DENV infection of human macrophages induces differential immunotranscriptomic profiles
Source: J Virol. 2025 Feb 4;99(3):e01962-24. doi: 10.1128/jvi.01962-24 (PMC11915858; doi:10.1128/jvi.01962-24)
Supplement: Figure S1 — Integrated UMAP projections of scRNAseq data split by sample origin. [file jvi.01962-24-s0001.pdf]

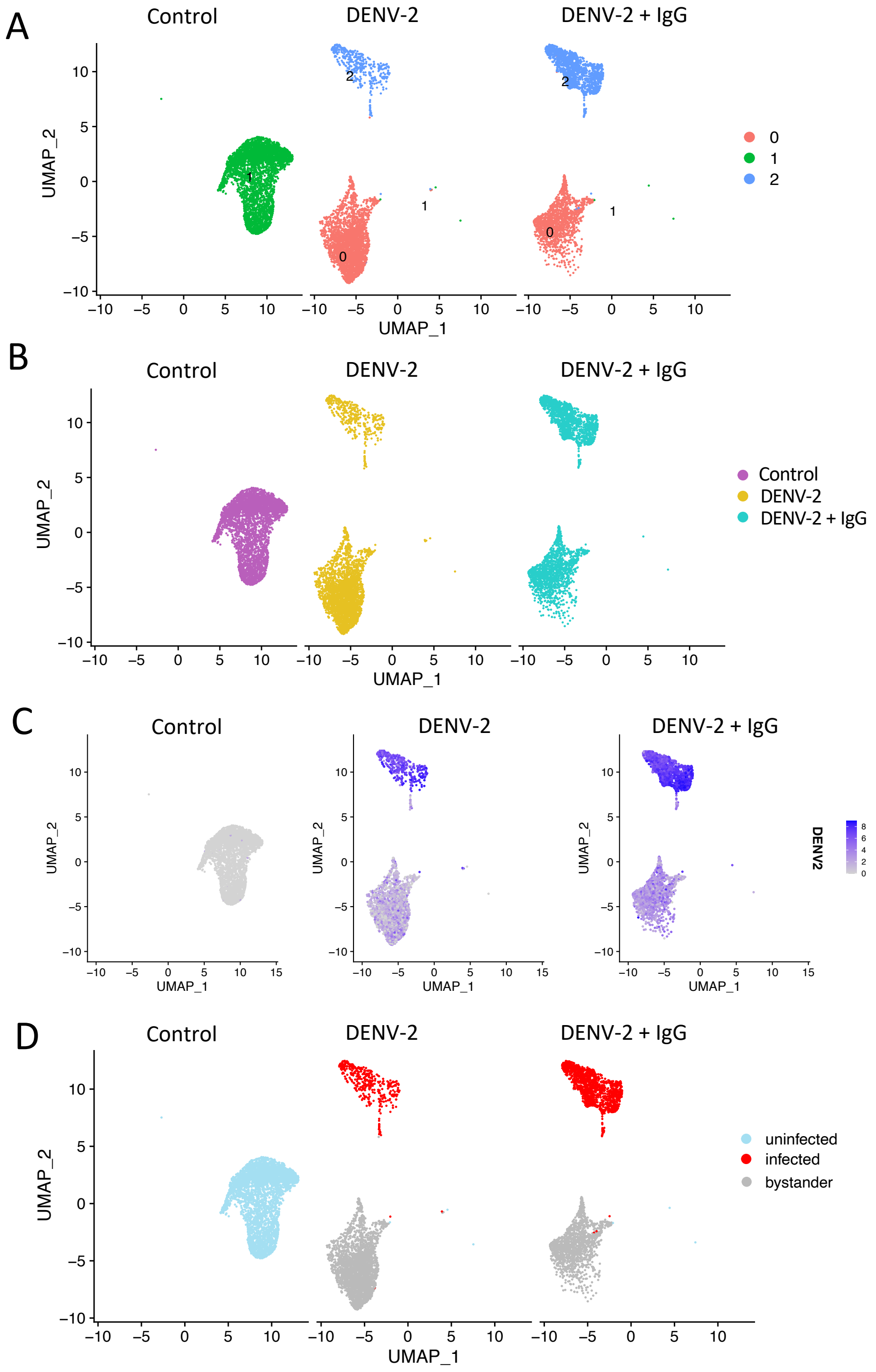

Figure S1. Integrated UMAP projections of scRNAseq data split by sample origin. (**A**) Seurat clusters (**B**) Sample origin (**C**) Feature plot of DENV positive sense RNA expression **(D)** Imputed infection state designation.
