# Supplementary material for: Conventional and antibody-enhanced DENV infection of human macrophages induces differential immunotranscriptomic profiles
Source: J Virol. 2025 Feb 4;99(3):e01962-24. doi: 10.1128/jvi.01962-24 (PMC11915858; doi:10.1128/jvi.01962-24)
Supplement: Figure S2 — DENV RNA expression across infection conditions. [file jvi.01962-24-s0002.pdf]

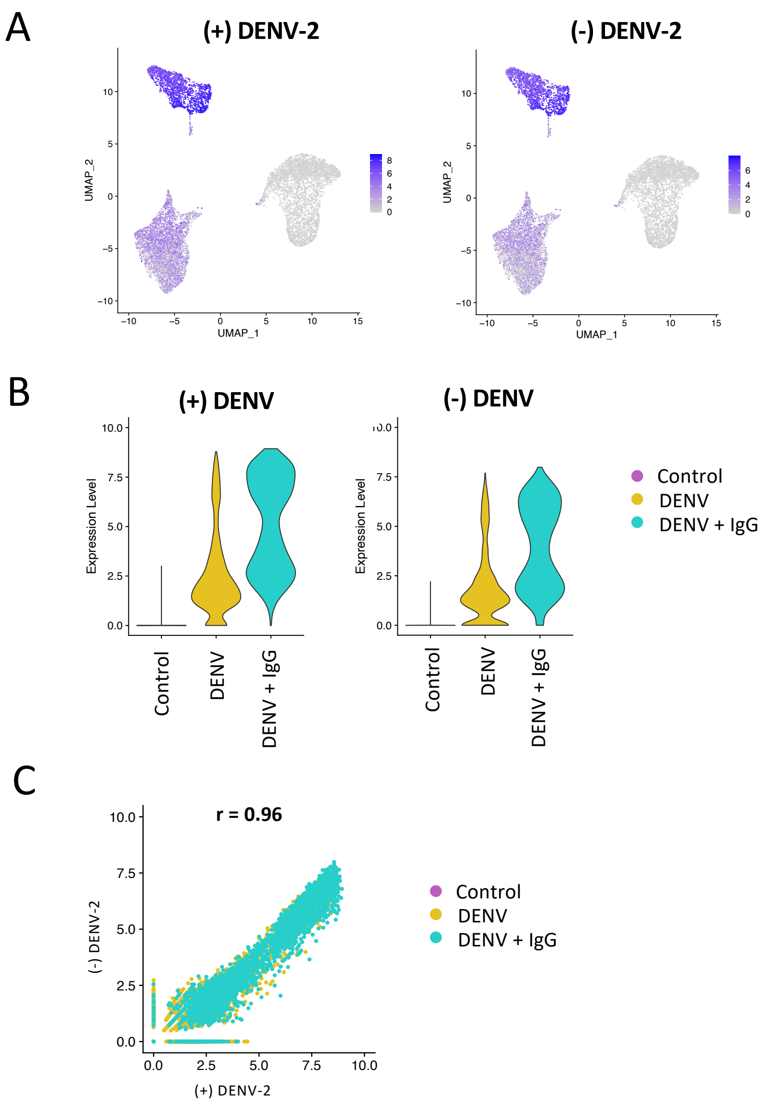

Figure S2. DENV RNA expression across infection conditions. (**A**) UMAP feature plots indicating (+) and (-) DENV RNA expression. **(B)** Violin plots of DENV2 positive (+) and negative (-) sense RNA expression across infection conditions. **(C)** Pearson correlation plot of DENV (+) and (-) sense RNA expression in infected cells.
