# Supplementary material for: Conventional and antibody-enhanced DENV infection of human macrophages induces differential immunotranscriptomic profiles
Source: J Virol. 2025 Feb 4;99(3):e01962-24. doi: 10.1128/jvi.01962-24 (PMC11915858; doi:10.1128/jvi.01962-24)
Supplement: Figure S3 — Pseudo-bulk gene expression and ingenuity pathway analysis of all macrophages in ADE compared to conventional infection conditions. [file jvi.01962-24-s0003.pdf]

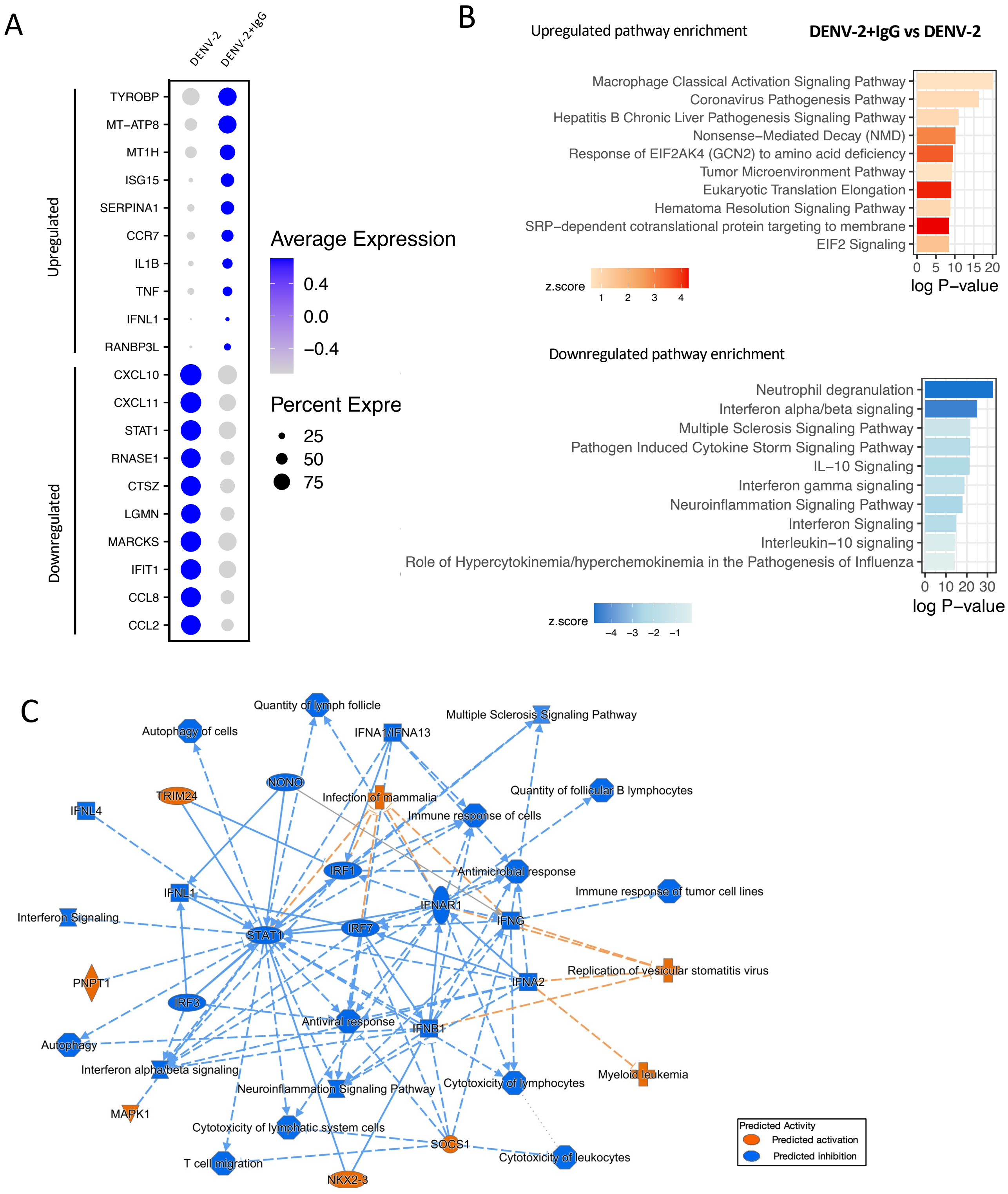

Figure S3. Pseudo-bulk gene expression and ingenuity pathway analysis of all macrophages in ADE compared to conventional infection conditions. (A) Dot plot highlighting selectively upregulated and downregulated DEGs in ADE compared to conventional infection, indicating average expression and percent of cells expressing a given transcript. **(B)** IPA based on DEGs between all cells in ADE compared to conventional infection. The top 10 upregulated and top 10 downregulated canonical pathways are displayed. **(C)** Graphical summary of IPA analysis in all macrophages in ADE compared to conventional infection conditions. In graphical summary plots, red colour indicates activation, while blue indicates suppression. Solid lines represent direct interactions, dashed lines represent indirect relationships, and dotted lines represent inferred relationships. Symbols represent the following: hourglass = canonical pathway, plus sign = disease, diamond = enzyme, square = cytokine, horizontal oval = transcriptional regulator, vertical oval = transmembrane receptor, octagon = function, circle = other, rectangle = G-protein coupled receptor, triangle = kinase.
